# Loss-of-function variants in JPH1 cause congenital myopathy with prominent facial and ocular involvement

**DOI:** 10.1136/jmg-2024-109970

**Published:** 2024-08-28

**Authors:** Mridul Johari, Ana Topf, Chiara Folland, Jennifer Duff, Lein Dofash, Pilar Marti, Thomas Robertson, Juan Vilchez, Anita Cairns, Elizabeth Harris, Chiara Marini-Bettolo, Khalid Hundallah, Amal M Alhashem, Mohammed Al-Owain, Reza Maroofian, Gianina Ravenscroft, Volker Straub

**Affiliations:** 1 Harry Perkins Institute of Medical Research, Centre for Medical Research, University of Western Australia, Nedlands, Perth, Western Australia, Australia; 2 Folkhälsan Research Center, Helsinki, Finland; 3 Department of Medical and Clinical Genetics, Medicum, University of Helsinki, Helsinki, Finland; 4 The John Walton Muscular Dystrophy Research Centre, Translational and Clinical Research Institute, Newcastle University and Newcastle Hospitals NHS Foundation Trust, Newcastle upon Tyne, UK; 5 Neuromuscular Research Group, IIS La Fe and CIBERER U763, Hospital Universitari i Politècnic La Fe, Valencia, Spain; 6 Anatomical Pathology, Queensland Pathology, Brisbane, Queensland, Australia; 7 School of Biomedical Sciences, University of Queensland, Brisbane, Queensland, Australia; 8 Neurosciences Department, Queensland Children's Hospital, Brisbane, Queensland, Australia; 9 Division of Pediatric Neurology, Department of Pediatric, Prince Sultan Military Medical City, Riyadh, Riyadh, Saudi Arabia; 10 Division of clinical genetic and metabolic medicine, Department of Pediatric, Prince Sultan Military Medical City, Riyadh, Saudi Arabia; 11 Department of Medical Genomics, Centre for Genomic Medicine, King Faisal Specialist Hospital and Research Centre, Riyadh, Saudi Arabia; 12 College of Medicine, Alfaisal University, Riyadh, Saudi Arabia; 13 Department of Neuromuscular Disorders, Queen Square Institute of Neurology, University College London, London, UK

**Keywords:** congenital, hereditary, and neonatal diseases and abnormalities, neuromuscular diseases, exome sequencing, RNA-seq

## Abstract

**Background:**

Weakness of facial, ocular and axial muscles is a common clinical presentation in congenital myopathies caused by pathogenic variants in genes encoding triad proteins. Abnormalities in triad structure and function resulting in disturbed excitation-contraction coupling and Ca^2+^ homeostasis can contribute to disease pathology.

**Methods:**

We analysed exome and genome sequencing data from four unrelated individuals with congenital myopathy characterised by facial, ocular and bulbar involvement. We collected deep phenotypic data from the affected individuals. We analysed the RNA-sequencing (RNA-seq) data of F3-II.1 and performed gene expression outlier analysis in 129 samples.

**Results:**

The four probands had a remarkably similar clinical presentation with prominent facial, ocular and bulbar features. Disease onset was in the neonatal period with hypotonia, poor feeding, cleft palate and talipes. Muscle weakness was generalised but prominent in the lower limbs with facial weakness also present. All patients had myopathic facies, bilateral ptosis, ophthalmoplegia and fatigability. Muscle biopsy on light microscopy showed type 1 myofiber predominance and ultrastructural analysis revealed slightly reduced triads, and structurally abnormal sarcoplasmic reticulum.

DNA sequencing identified four unique homozygous loss-of-function variants in *JPH1*, encoding junctophilin-1 in the four families; one stop-gain (c.354C>A;p.Tyr118*) and three frameshift (c.373delG;p.Asp125Thrfs*30, c.1738delC;p.Leu580Trpfs*16 and c.1510delG;p. Glu504Serfs*3) variants. Muscle RNA-seq showed strong downregulation of *JPH1* in the F3 proband.

**Conclusions:**

Junctophilin-1 is critical for the formation of skeletal muscle triad junctions by connecting the sarcoplasmic reticulum and T-tubules. Our findings suggest that loss of *JPH1* results in a congenital myopathy with prominent facial, bulbar and ocular involvement.

**WHAT IS ALREADY KNOWN ON THIS TOPIC**

- Previous studies have shown that pathogenic variants in genes encoding triad proteins lead to various myopathic phenotypes, with clinical presentations often involving muscle weakness and myopathic facies.
- The triad structure is essential for excitation-contraction coupling and Ca^2+^ homeostasis and is a key element in muscle physiology.

**WHAT THIS STUDY ADDS**

- This study identified novel homozygous loss-of-function variants in the *JPH1* gene, linking them to a form of congenital myopathy characterised by severe facial and ocular symptoms.
- Our research sheds light on the critical impact on junctophilin-1 function in skeletal muscle triad junction formation and the consequences of its disruption resulting in a myopathic phenotype.

**HOW THIS STUDY MIGHT AFFECT RESEARCH, PRACTICE OR POLICY**

- This study establishes that homozygous loss-of-function mutations in *JPH1* cause a congenital myopathy predominantly affecting facial and ocular muscles.
- This study also provides clinical insights that may aid the clinicians in diagnosing similar genetically unresolved cases.

## Introduction

 In skeletal muscles, the sarcoplasmic reticulum (SR) is surrounded by specialised invaginations of the sarcolemma in the form of terminal cisternae and transverse tubules (T-tubules). The juxtaposition of a T-tubule with two terminal cisternae forms the triad.^1^ In the triads, proteins, notably the dihydropyridine receptor (DHPR) in the T-tubule and the ryanodine receptor (RYR) in the SR, maintain Ca^2+^ homeostasis and are crucial in excitation-contraction (EC) coupling.^2^

Disturbed EC coupling and Ca^2+^ homeostasis, along with secondary abnormalities, including structural alterations of T-tubules, triad structure and function^2 3^ are the pathomechanisms of myopathies associated with variants in genes encoding proteins critical to EC coupling, including *RYR1*, *CACNA1S*, *ORAI1*, *STAC3*, *STIM1*, *MTM1*, *DNM2, TRDN* and *BIN1*. These disorders are collectively referred to as triadopathies.^3^

**Figure 1:**
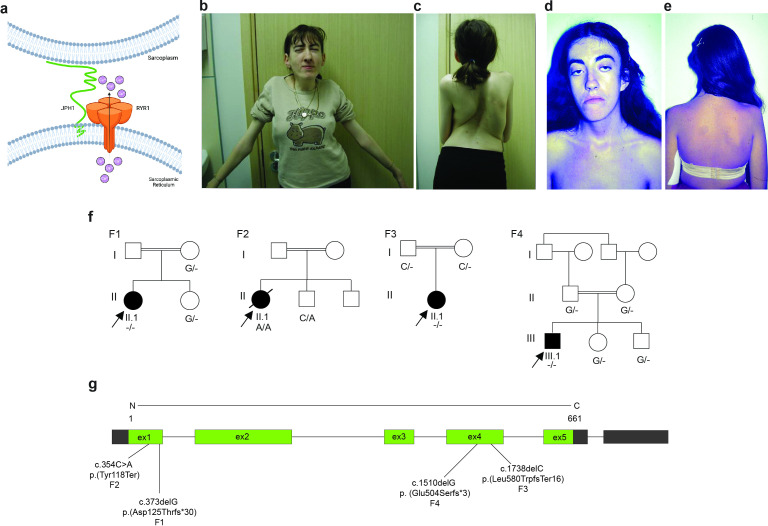
*JPH1*-related myopathy in four families. (A) Schematic representation of interaction of junctophillin-1 (JPH1) and ryanodine receptor type 1 (RYR1) at the neuromuscular triad. Flow of Ca^2+^ is indicated from the sarcoplasmic reticulum to the sarcoplasm through RYR1. (B) Bilateral ptosis and ophthalmoplegia and (C) kyphoscoliosis in patient F1-II.1. (D) Facial weakness and ophthalmoplegia and (E) dorsal scoliosis, lumbar lordosis and winged scapulae in patient F2-II.1. (F) Pedigrees of the four consanguineous families included in this study, family 1 and 2 are of European origin, family 3 is of Khmer origin and family 4 is of Middle Eastern origin. Genotypes are shown for the identified *JPH1* variants. (G) A scheme of identified pathogenic variants in *JPH1* and their position on the *JPH1* gene model.

Junctophilins are key proteins responsible for triad structure formation and maintenance in striated muscle.^4^ There are three junctophilin genes. *JPH1* is predominantly expressed in skeletal muscles, while *JPH2* is expressed in cardiac and skeletal muscles and *JPH3* specifically in the brain.^5 6^ In the skeletal muscle triad, JPH1 interacts with RYR1 aiding in the release of Ca^2+^ (figure 1A). In vitro, downregulation or loss of junctophilins can result in defective triads and dysregulated Ca^2+^ homeostasis due to mislocalisation of RYR1 and DHPR.^7 8^ *Jph1* knockout (KO) mice die shortly after birth, with ultrastructural analysis showing defective and reduced triads along with structurally abnormal SR.^4^

Here, we report four unrelated probands with strikingly similar phenotypes involving facial and ocular muscle weakness caused by homozygous null variants in *JPH1*. Our deep phenotyping and novel genetic findings expand the spectrum of congenital myopathies caused by defects in triad proteins and provide evidence for the first time that loss of *JPH1* results in a skeletal muscle disease and should be classified as a triadopathy.

## Methods

### Patients and clinical examinations

Blood samples were collected from four unrelated affected patients and seven additional asymptomatic family members. Consanguinity was known, or suspected, for all the families. Patients’ biomaterials for diagnostic purposes were collected after written informed consent was obtained from the patients or their legal guardians by the referring clinicians.

All four probands underwent clinical neuromuscular examination. Ancillary tests, including electrophysiological examinations (nerve conduction studies and needle electromyogram) and serum creatine kinase levels were obtained in all patients.

### Molecular genetics

Genomic DNA was isolated from blood cells of probands and available family members, using standard techniques.

Exome sequencing (ES) from the genomic DNA of F1-II.1 and F2-II.1 and F4-III.1 was carried out by the Broad Institute Genomics Platform using an 8 MB targeted Illumina exome capture. PCR-free libraries were prepared from the genomic DNA of F3-I.1, F3-I.2 and F3-II.1. Short-read (sr) genome sequencing (GS) was performed on NovaSeq 6000 (Illumina, San Diego, California, USA) with pair-end 150 bp reads at the Kinghorn Centre for Clinical Genomics (Garvan Institute of Medical Research, New South Wales, Australia).

Single nucleotide variant (SNV) analysis for the four families was performed using *seqr*,^9^ hosted by the Centre for Population Genomics, a collaboration between Garvan Institute of Medical Research (Sydney, Australia) and the Murdoch Children’s Research Institute (Melbourne, Australia).

ES and srGS results were analysed and SNV/indels were filtered using a minor allele frequency ≤0.0001 in the Genome Aggregation Database V.2.1.1 (hg19) and V.3.1.2 (hg38).

Variants in *JPH1* are annotated on NM_020647.2 and NP_065698.1. All identified variants were also evaluated for current American College of Medical Genetics and Genomics (ACMG) pathogenicity annotations using VarSome,^10^ Alamut (Alamut Visual Plus V.1.6.1, SOPHiA GENETICS) and Mutalyzer.^11^

### Muscle biopsy, immunohistochemical and imaging studies

Snap-frozen muscle biopsy samples were obtained from three affected patients (F1-II.1: quadriceps, F2-II.1: deltoid, F3-II.1: right upper arm). Routine muscle histopathological studies were performed, including H&E, modified Gomori’s trichrome and NADH tetrazolium reductase staining.^12^ DAB immunostaining was performed using mouse monoclonal antimyotilin (clone RSO34, 1:20, LEICA Biosystems Newcastle, UK) and mouse monoclonal antidesmin (clone D33, 1:70, Richard-Allan Scientific, USA), with Mouse ExtrAvidin Peroxidase Staining Kit (EXTRA2, Merck KGaA, Darmstadt, Germany). Microscopic images were obtained using a NIKON ECLIPSE Ci microscope equipped with an OLYMPUS ColorView II camera.

For patient F3-II:1, ultrathin resin sections with a thickness of 70–80 nm were prepared for electron microscopy and examined with an FEI Morgagni 268 Transmission Electron Microscope operating at 80 kV. Electron micrographs were obtained using the Olympus-SIS Morada digital camera (Olympus Soft Imaging Solutions, Münster, Germany).

### RNA-sequencing

Total RNA was extracted from patient F3-II:1 and control skeletal muscle biopsies (~15–50 mg) using the RNeasy Fibrous Tissue Mini Kit (Qiagen, Hilden, Germany) according to the manufacturer’s instructions. Strand-specific Poly-A+RNA libraries were prepared from extracted RNA using the Agilent SureSelect XT library preparation kit (Agilent, Santa Clara, California, USA). QC was performed using TapeStation 4200 (Agilent) and Qubit 4 Fluorometer (Thermo Fischer Scientific, Waltham, Massachusetts, USA), as well as QC sequencing on an Illumina iSeq 100 flowcell (Illumina, San Diego, California, USA). These strand-specific libraries were sequenced on an Illumina NovaSeq 6000 to produce paired-end 150 bp reads and an average of 50 million read pairs per sample. Adaptor sequences were removed and demultiplexed FASTQ files were provided by Genomics WA (Western Australia) for download and further analysis. FASTQ files were processed, including read quality control and alignment, using the nf-core/rnaseq pipeline (https://nf-co.re/rnaseq), V.3.8.1. Trimmed reads were aligned to the NCBI GRCh38 human reference genome using STAR V.2.7.10a^13^ (STAR, RRID: SCR_004463). We used DROP V.1.0.3,^14^ as previously described^15^ to analyse aberrant gene expression among a cohort of 129 skeletal muscle RNA-sequencing (RNA-seq) datasets from rare muscle disease patients and unaffected controls. DROP leverages OUTRIDER,^16^ which uses a denoising autoencoder to control co-variation before fitting each gene over all samples via negative binomial distribution. Multiple testing correction was done across all genes per sample using DROP’s in-built Benjamini-Yekutieli’s false discovery rate method. Plots were prepared using R (V.4.1.3) in RStudio. The splicing pattern and expression of *JPH1* in F3 was visualised using Integrative Genomics Viewer (IGV)^17^ and plots were created using ggsashimi.^18^

### Data sharing statement

ES and srGS data of probands and family members are available on *seqr*. All relevant clinical data are shared as part of this study.

Identified variants in *JPH1* have been submitted to ClinVar with accession numbers SCV004228294–SCV004228296 and SCV004697810.

Code for generating plots is available at: https://github.com/RAVING-Informatics/jph1-cm

## Results

### Clinical findings in patients with *JPH1*-related myopathy

The clinical findings of all four probands are summarised in table 1. In general, the four probands had a remarkably similar presentation with global distribution of muscle weakness and generalised muscle wasting, with F3-II.1 notably exhibiting thin muscle bulk. They showed facial weakness accompanied by bilateral ptosis and ophthalmoplegia (patient F1-II.1, figure 1B; patient F2-II.1, figure 1D), a nasal voice and dysphagia. They also presented with myalgia, exercise intolerance and fatigability. Reduced forced vital capacity was prominent in F1-II.1 (19% of predicted value), who needed non-invasive ventilation. The patients also showed kyphoscoliosis (patient F1-II.1, figure 1C) lordosis and scoliosis (patient F2-II.1, figure 1E). None of the patients showed any cardiac involvement or intellectual impairment. First clinical assessments indicated either a novel congenital myopathy or a congenital myasthenic syndrome-like phenotype.

**Table 1:** Clinical, histopathological and MRI details of patients included in the study

Patient ID	F1-II.1	F2-II.1	F3-II.1	F4-III.1
*JPH1* variant	c.373delG, p.(Asp125Thrfs*30)—homozygous	c.354C>A, p.(Tyr118*)—homozygous	c.1738delC, p.(Leu580Trpfs*16)—homozygous	c.1510delG, p.(Glu504Serfs*3)—homozygous
Current age/age at last exam	Fourth decade/Fourth decade	Deceased/Fifth decade	Second decade/Second decade	First decade/First decade
Age of onset	Congenital	Congenital	Congenital	Congenital
Motor development	Not sitting at 11 months	Delayed motor milestones—walked at 3 years	Delayed motor milestones—assisted standing at 20 months, walked at 2.5 years	Delayed motor milestones—assisted standing at 20 months, walked at 4 years
Progression	Slowly progressive	Stable through childhood, slow decline since fourth/fifth decade	Relatively stable through childhood	Relatively stable
Maximum motor ability	Walking independently for up to 10 min	Able to walk unaided, able to climb stairs with aid of railing	Current motor ability	Walk alone
Motor ability	Mainly manual wheelchair user, able to stand up with assistance, unable to walk	Able to stand up with assistance, able to walk 10 m with support	Able to walk around school, able to run (but slowly). Able to climb stairs with railings. NSAA=31/34	Motor delay. Able to walkAble to climb stairs with railings
Muscle strength (MRI scale)	No recent assessment	Scapular 3/5, hip girdle 3/5 and distal UL and LL 4-/3. Unable to lift upper limbs to head, cannot tip-toe or stand on heels	Hip girdle 4-/5, distal UL 4+/5, 4/5 power elsewhere, 1–2 handed Gowers	Severe peripheral hypotonia and distal weakness
Distribution of weakness	Generalised (proximal and distal, upper and lower limbs), including facial weakness and ptosis	Generalised (proximal and distal upper and lower limbs), including ptosis and facial and neck flexor weakness	Generalised with ptosis and facial diplegia, no neck flexor weakness	Generalised with ptosis, no neck flexor weakness
Reflexes	Absent	Absent	Absent in LL; reduced in UL	Absent
Contractures	Talipes at birth	Talipes at birth; ankle contractures	No—hypermobile at ankles	N/A
Spine involvement	Kyphoscoliosis, surgery at 12 years old, rigid spine (thoraco-lumbar)	Dorsal scoliosis, lumbar lordosis and winged scapulae	Mild lumbar lordosis; no scoliosis	Mild scoliosis
Muscle wasting	Generalised wasting	Generalised wasting	Generalised wasting; thin muscle bulk	Generalised wasting
Ocular symptoms	Bilateral ptosis and ophthalmoplegia	Ptosis and ophthalmoparesis (surgical correction of ptosis and squint in childhood)	Ptosis and ophthalmoplegia with mild limitation of upward gaze	Ptosis and ophthalmoplegia with mild limitation of upward gaze
Bulbar symptoms	Dysphagia	Nasal voice, no dysphagia	Mild dysarthria, short-term NGT feeds at birth	Mild dysarthria, NGT feeding, now GT feeding
Respiratory involvement	Respiratory insufficiency at birthFVC=0.5 L (19%). Non-invasive ventilation (BiPAP)	No respiratory support. FVC=1.76 L (63%). PIMmax: 43%, PEMmax: 72%	No respiratory support. FVC=1.22 L (57%), FEV1=1.20 (62%). Normal sleep study	No respiratory support
Other	Cleft palate, migraine	Cleft palate. Myalgia and exercise intolerance. Hypoacuasia in the fourth decade (neural deafness). Alcoholic liver cirrhosis, died from hepatic insufficiency (transplant excluded)	Exercise intolerance with fatigue	Left vocal cord palsy. Bilateral coxa valga, generalised osteopenia. Bilateral undescended testes status postorchidopexy dysmorphic, microcephaly, high arched palate, developmental delay, GERD, swallowing incoordination and constipation
EMG findings	Mild myopathic changes (in childhood)	Myopathic features. Normal RNS and jitter	N/A	N/A
Muscle histopathology	Biopsy at 11 years, quadriceps.Type 1 myofiber predominance, with <2% of type myofibers. Some hypertrophied myofibers and disseminated atrophic myofibers of both types	Biopsy at 38 years, deltoid. Type 1 myofiber predominance, few centralised nuclei. Occasional hypertrophic myofibers present and scattered hypotrophic type 2 myofibers. Mild focal fibrosis. No myofiber degeneration or regeneration, nor abnormalities in NADH-SDH oxidative reactions	Biopsy of upper arm. Type 1 myofiber predominance and mostly type 2 myofiber atrophy. No grouping of myofiber types	N/A
EM findings	N/A	N/A	Slightly reduced triads, occasional small minicore-like foci	N/A

BiPAPBi-level Positive Airway PressureEMelectron microscopyFEV1forced expiratory volume in 1 sFVCforced vital capacityGERDGastroesophageal reflux diseaseLLlower limbN/Anot assessedNADH-SDHnicotinamide adenine dinucleotide-succinate dehydrogenaseNGTnasogastric tubeNSAANorth Star Ambulatory AssessmentPEMAXMaximal expiratory mouth pressuresPIMAXMaximal inspiratory mouth pressuresRNSrepeat nerve stimulationULupper limb

### Identification of deleterious variants in *JPH1*

Analysis of ES (F1, F2 and F4) and srGS (F3) data were initially negative for approximately 600 genes known to cause a neuromuscular phenotype.^19 20^ Subsequently, we identified four unique homozygous protein truncating variants in *JPH1*: two in exon 1, c.373delG, p.(Asp125Thrfs*30) and c.354C>A, p.(Tyr118*), in F1 and F2, respectively and two in exon 4, c.1738delC, p.(Leu580Trpfs*16) and c.1510delG; p.(Glu504Serfs*3) in F3 and F4, respectively (figure 1F, G, online supplemental figure). Using VarSome and Alamut, we assessed the pathogenicity of the identified variants. Since all four variants would result in null alleles and were absent in the reference population databases, they fulfilled the PVS1 (very strong) and PM2 (supporting) criteria of the ACMG guidelines, resulting in classification of the variants as ‘likely pathogenic/pathogenic’.

### Muscle pathology associated with biallelic loss-of-function *JPH1* variants

**Figure 2:**
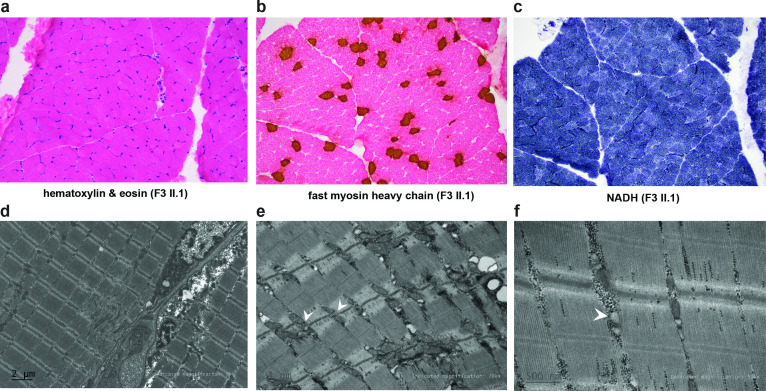
Muscle pathology of patient F3-II.1. (A) H&E showing preserved muscle structure. (B) Immunohistochemistry for fast myosin heavy chain (stained with DAB (brown)) and eosin showing type 1 myofiber predominance. Most of the atrophic myofibers stain as type 2 but the normal chequerboard distribution of fibre types appears relatively preserved (C) NADH staining. No cores, or minicores are present. Electron microscopy (patient F3-II.1) showing some focal Z-band streaming. The observed area of Z-line streaming is near the sarcolemma, this finding in this instance may be non-specific (D); reduced triads with dilated sarcoplasmic reticulum (white arrowheads) (E,F).

Muscle biopsies from patients F2-II.1 and F3-II.1 revealed a striking pattern of type 1 myofiber predominance (figure 2A). No other characteristic features were observed; there was no increase in internally or centrally located nuclei (figure 2A), or staining suggestive of cores(figure 2C) or nemaline bodies. Electron microscopy analysis of the muscle biopsy of F3-II.1 showed ultrastructural defects including some focal and possibly non-specific Z-band streaming, slightly reduced number of triads and structurally abnormal SR which appeared dilated (figure 2D–F).

### Analysis of skeletal muscle RNA-seq data from proband F3-II.1

**Figure 3:**
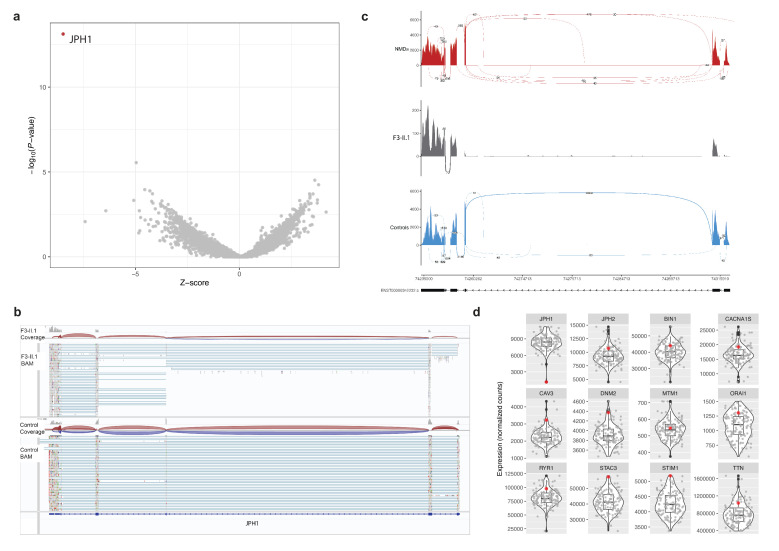
Splicing and gene-expression in skeletal muscle from a patient with *JPH1*-related myopathy. (A) Volcano plot showing results from the OUTRIDER analysis. *JPH1* is indicated as an outlier in red colour. (B) Visualisation of RNA-sequencing (RNA-seq) data in Integrative Genomics Viewer comparing *JPH1* expression with a control. (C) *ggsashimi* plot analysis of *JPH1* from RNA-seq data of F3-II.1. *JPH1* expression and splicing patterns of the patient are shown in grey colour, compared with other NMD patients in red (n=39) and unaffected controls in blue (n=6). (D) Normalised expression of genes encoding for other triad proteins is presented as box plots. Median and quartile values are shown, with whiskers reaching up to 1.5 times the IQR. Expression levels from individual samples in the cohort are shown with jitter points and that of F3-II.1 is represented with red colour. The violin plot illustrates the distribution of data in each cohort. The scaled Y-axis shows normalised counts. NMD, neuromuscular disease.

OUTRIDER analysis detected under expression of *JPH1* as an outlier (figure 3A) in F3-II.1 (Z=−8.48, p-adj=1.15×10^–8^). Based on normalised gene counts, *JPH1* expression in F3-II.1 was the lowest at 1167.87 compared with the other 129 patients with muscle disease and healthy controls (log_2_fold change=−2.84). IGV analysis (figure 3B) and sashimi plots (figure 3C) confirmed the low expression of the gene.

Since, a reduced expression of *JPH1* could affect other T-tubule proteins, we additionally analysed the expression of other genes encoding components of the triad and T-tubules, including: *RYR1*, *CACNA1S*, *JPH2*, *MTM1*, *DNM2*, *BIN1*, *STAC3*, *ORAI1*, *STIM1*, *CAV3* and *TTN* (figure 3D). There were no differences in expression levels for any of these genes of interest in *JPH1*-related myopathy compared with healthy control muscle or patients with other forms of neuromuscular diseases.

## Discussion

Our results demonstrate that loss-of-function variants in *JPH1*, coding for junctophillin-1, result in a congenital myopathy, characterised by global distribution of muscle weakness and wasting, but with prominent facial muscle weakness, bilateral ptosis, exercise intolerance and fatigability.

In skeletal muscles, junctophilins have a regulatory and maintenance function with other triad proteins, including assembly of Ca^2+^ release complex and organisation of the Store Operated Ca^2+^ entry pathway through interaction with other T-tubule proteins including RYR1, DHPR and CAV3.^2^

All four probands showed prominent myalgia, along with exercise intolerance and fatigability. These features are commonly seen in other triadopathies, such as tubular aggregate myopathies caused by pathogenic variants in *STIM1*.^3^

In our patients, we observed homozygous null variants in *JPH1* resulting in no expression of complete transcript suggesting no viable production of JPH1. This is well reflected in our morphological and ultrastructural studies which concur with *Jph1* KO mice. EM analysis of muscle biopsy of F3-II.1 showed a reduced number of triads. Light microscopy analysis of F2-II.1 and F3-II.1 showed predominance of type 1 myofibers. Generally, triad abundance varies in different myofiber types in skeletal muscle due to the distinct Ca^2+^ requirements of EC coupling of the functionally different myofiber types. Myofibers under higher contraction load require more triads due to the greater and faster Ca^2+^ influx and efflux requirements. Type 1 myofiber predominance is also observed in other triadopathies, including *RYR1*, *DNM2*, *BIN1* and *MTM1*-associated congenital myopathies.^21^,^24^

EM analysis of muscle biopsy of F3-II.1 also showed dilated SR. Disorganisation of triads and swelling of SR was observed in mutant muscles of *Jph1* KO mice.^4^ The swelling of SR can be attributed to SR Ca^2+^ overloading and has been seen in mice lacking both *ryr1* and *ryr3*.^25^ Likewise, in human muscles lacking JPH1, SR Ca^2+^ overloading could cause similar abnormalities due to reduced triad junctions potentially hindering DHPR-mediated activation of RYR. Further characterisation of the spectrum of pathologies associated with *JPH1*-related myopathy will be needed as additional patients are identified.

Congenital myopathies arising due to pathogenic variants in genes encoding components of the triads or proteins involved in triad formation and maintenance, including RYR1 and STAC3, share many clinical features.^26 27^ These include hypotonia and axial weakness, which often tends to be static or slowly progressive, facial and bulbar weakness, resulting in dysphagia and dysarthria, ocular weakness, including ptosis and ophthalmoplegia and respiratory insufficiency. Joint contractures may be present at birth.^26 27^

Previously, deficiency of Jph1 in mouse models was shown to result in neonatal death. This was attributed to failure in suckling, as a newborn due to weak contractile activity of jaw muscles and weak pharyngo-oesophageal or diaphragm muscles.^4^ The myofibers of these *Jph1* KO mice were morphologically normal, and analysis of muscle histology did not detect obvious abnormalities. Ultrastructural analysis using electron microscopy, however, revealed that *Jph1* KO neonates had swollen SR and defective and highly reduced triads. These observations suggested that loss of JPH1 clearly affects triad formation in skeletal muscles.^4^

Additionally, reduced JPH1 expression has been associated with defective triad formation and disturbed Ca^2+^ homeostasis due to mislocalisation of RYR1 and DHPR.^7 8 28^ While the overall disease presentation is similar in *Jph1* KO mice and *JPH1* patients, none of our patients had severe muscle weakness or a dystrophic phenotype as seen in neonatal mice.

Analysis of RNA-seq data showed that mRNA expression of other key genes of the triad are unaltered in *JPH1* patient’s skeletal muscle compared with healthy controls and other neuromuscular disease biopsies. This is perhaps not surprising given the relatively mild phenotype observed in these patients, compared with affected individuals with bi-allelic loss-of-function variants in *CACNA1S, RYR1* or *STAC3*.

This would suggest that the loss of JPH1 observed in our patients due to homozygous null variants, affects the triad formation and maintenance. The exact pathomechanism of how loss of JPH1 and normal expression of other triad genes contribute to the phenotype, remains to be understood.

Our results, show for the first time that bi-allelic null variants in *JPH1* cause a congenital myopathy characterised by prominent facial and ocular muscle weakness. Hence, *JPH1* should be included in genetic screenings of unsolved patients with similar clinical presentation.

## supplementary material

10.1136/jmg-2024-109970 online supplemental file 1

## Data Availability

Data may be obtained from a third party and are not publicly available. All data relevant to the study are included in the article or uploaded as supplementary information.
